# Implementing evidence based health skills in practice through higher education

**DOI:** 10.1186/1472-6963-14-S2-P38

**Published:** 2014-07-07

**Authors:** Eva Ekvall-Hansson, Gunilla Carlsson, Agneta Malmgren Fänge

**Affiliations:** 1 Dept of Clinical Sciences, Lund University, Lund, Sweden; 2 Dept of Health Sciences, Lund University, Lund, Sweden

## 

The promotion of a sustainable evidence-based health care practice requires multiple efforts. Since master’s level courses in health sciences in Sweden target practicing professionals, integrating knowledge and skills related to evidence-based practice into higher education is one way of reaching this goal. Here we describe the implementation of the course Evidence Based Practice, 7.5 ECTS at the Master of Medical Sciences program at Lund University, Sweden. The course is elective and enrolls registered nurses, physiotherapists and occupational therapists, i.e. the main fields. It is internet based, with five seminars on campus. The learning activities are interprofessional, with each student taking a bearing on their main field. The mode of teaching applied is Targeting Specific Skills of Evidence Based Practice [1].

Examples of learning outcomes are:

Independently identify, explain and discuss methods for developing evidence-based practice; independently identify problem areas and formulate appropriate clinical questions (CQ); identify, critically review and discuss evidence-based knowledge in healthcare.

Besides lectures and seminars the students have four mandatory assignments:

1. Define 3-4 relevant CQ for the course.

2. Review the literature related to the CQ above, and classify two papers according to the PICO map [1].

3. A clinical guideline is critically reviewed and summarized according to a checklist [2].

4. A web-site focusing on health information for lay people is critically reviewed and summarized.

Teacher feedback is given throughout.

The examination is an individual paper. Following the PICO map CQs are identified and formulated. The literature is systematically reviewed and the five papers with the highest evidence are critically appraised and summarized, including a popular science abstract. At the final seminar the students present and defend the paper orally, including an opposition on another student’s paper. In their evaluations the students express above all that they search and review scientific papers more systematically and critically. They state that they now know how to frame CQs and that they apply the acquired knowledge and skills in professional practice. The implementation of this type of course into higher education is a feasible way to enhance the use of evidence based knowledge and skills in health care practice.
